# Human iPSC-derived 3D cardiac models for cardiomyopathies: organoids, spheroids, and engineered heart tissues as translational platforms

**DOI:** 10.1093/stmcls/sxag039

**Published:** 2026-07-08

**Authors:** Prabin Upadhyaya

**Affiliations:** Division of Cardiology, Department of Medicine and Surgery, Università degli studi di Milano-Bicocca, Milan 20126, Italy; Experimental Laboratory for Genetic-Based Cardiac Arrhythmia Research, IRCCS Istituto Auxologico Italiano, Cusano Milanino 20095, Italy

**Keywords:** iPSCs, spheroids, EHTs, cardiac organoids, disease modeling

## Abstract

Cardiomyopathies represent a substantial global health burden, yet progress in developing effective therapies was long constrained by the absence of physiologically relevant human models to understand molecular mechanisms and evaluating interventions. The advent of induced pluripotent stem cell (iPSC) technology has driven major advances in cardiomyopathy research, although important limitations persist. Current iPSC-based systems exhibit incomplete cellular maturation, lack functional vasculature and chamber level architecture, and possess an immature extracellular matrix. These factors must be carefully weighed when interpreting disease phenotypes and drug response data. Induced pluripotent stem cell-derived in vitro three-dimensional (3D) cardiac tissues have significantly addressed these issues, thereby expanding our ability to model human cardiac disease, offering more physiologically relevant platforms for mechanistic studies, drug screening, and translational research. This concise review synthesizes recent advances in the development of iPSC-derived 3D cardiac systems, such as 3D spheroids, engineered heart tissues, and cardiac organoids, their application to modeling various cardiomyopathies including hypertrophic cardiomyopathy, dilated cardiomyopathy, and amyloid-related cardiomyopathies, while discussing critical challenges including cellular maturation, vascularization, and standardization. In this review, emerging technologies and therapeutic strategies have been highlighted that bridge the gap between bench and bedside, demonstrating the translational potential of these models for precision medicine in cardiovascular disease.

## Graphical Abstract

**Graphical Abstract:**
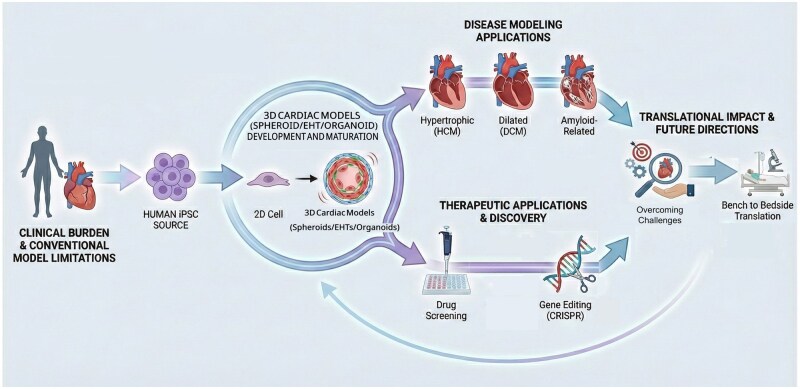

Significance statement Cardiomyopathies cause significant morbidity and mortality worldwide, yet therapeutic development is hindered by inadequate human disease models. This comprehensive review synthesizes advances in induced pluripotent stem cell-derived cardiac three-dimensional (3D) disease models, demonstrating their transformative potential for cardiovascular research. The sophisticated 3D models successfully recapitulate patient-specific disease phenotypes, enabling mechanistic investigation, high-throughput drug screening, and gene editing approaches impossible with conventional systems. By bridging the translational gap between animal models and human disease, cardiac models are revolutionizing precision medicine approaches for genetic cardiomyopathies. This review provides critical insights for researchers, clinicians, and pharmaceutical developers advancing therapeutic strategies from bench to bedside in cardiovascular medicine.

## Introduction

### Clinical burden of cardiomyopathies

Cardiomyopathies constitute a heterogeneous group of myocardial disorders affecting an estimated 10 million individuals worldwide and represent a major cause of heart failure, arrhythmias, and sudden cardiac death, particularly among younger and working-age populations.^1^ The prevalence of cardiomyopathies is estimated at approximately 1:250-1:500 individuals for common forms such as dilated and hypertrophic cardiomyopathy, with a substantial proportion attributable to genetic causes.^1–3^ In the United States, dilated cardiomyopathies (DCM) contribute significantly to the burden of heart failure and are a leading indication for advanced therapies including transplantation and mechanical circulatory support.^3^

In broader heart failure cohorts, 1-year case fatality has been reported to range from 4% to 45%, with a pooled average of approximately 33%. Dilated cardiomyopathy, in particular, is often progressive and may lead to advanced heart failure and death in the absence of definitive therapy.^4^ Device-based therapies such as implantable cardioverter-defibrillators and cardiac resynchronization therapy reduce arrhythmic risk and improve cardiac function but do not address the underlying disease biology.^5^^,^^6^ For patients with advanced disease, left ventricular assist devices serve as bridge-to-transplant or destination therapy, although their use is limited by significant complications.^7^ Cardiac transplantation remains the definitive treatment but is severely constrained by donor organ scarcity, restricting access to a small proportion of eligible patients.^7^^,^^8^ Moreover, lifelong immunosuppression exposes recipients to infection, malignancy, and allograft vasculopathy.^9^^,^^10^ Collectively, these limitations point to an urgent need for human disease models that can reveal truly disease-modifying therapies and reliably predict individual disease trajectories and treatment responses, while accurately reflecting the complexity of cardiac physiology and the substantial genetic and phenotypic heterogeneity of cardiomyopathies. Traditional research models, including transgenic animals and heterologous cell cultures, have provided valuable insights but often fail to fully recapitulate the complexity and specificity of human cardiac pathophysiology.

### Limitations of conventional disease models

Animal models have historically served as essential tools for understanding cardiac disease mechanisms and evaluating potential therapeutics. However, these models present significant limitations including species-specific differences in cardiac physiology, electrophysiology, and drug metabolism that often do not translate effectively to humans.^11^ Two-dimensional (2D) monolayer cell cultures, while useful for initial mechanistic investigations, lack the cellular complexity, tissue-level organization, and cell-cell interactions that characterize native cardiac tissue.^11^ The consequences of these translational gaps are well documented in several recent high-profile clinical failures. Gene-based enhancement of SERCA2a, which consistently improved calcium handling and contractility in rodent and large-animal models, showed no clinical benefit in the CUPID 2 trial despite robust target engagement.^12^ Antioxidant therapies such as *N*-acetylcysteine, long believed to mitigate oxidative stress and remodeling in experimental systems, similarly produced inconsistent or largely neutral effects on clinical endpoints in randomized cardiovascular trials and meta-analyses.^13^ More recently, mavacamten, a cardiac myosin inhibitor with strong mechanistic rationale and encouraging phase 2 biomarker signals in nonobstructive hypertrophic cardiomyopathy, failed to improve functional capacity or patient-reported health status in the phase 3 ODYSSEY-HCM trial.^14^ Long-acting relaxin-pathway activation provides another recent example of mechanistic and early translational promise failing to translate into clinical benefit: volenrelaxin, a long-acting human relaxin analogue intended to improve congestion, renal function, and atrial dysfunction in HFpEF, improved left atrial reservoir strain only at the lowest dose but was associated with worsening congestion signals, higher NT-proBNP, and a nonsignificant increase in heart-failure hospitalization risk in a 2025 randomized phase 2 trial.^15^ Likewise, stem cell therapies that appeared regenerative in murine infarction models have yielded inconsistent and often neutral in larger rigorously controlled studies.^16^ These persistent translational gaps reflect fundamental physiological and molecular differences between species, including divergent myosin isoform expression, calcium-cycling kinetics, electrophysiological properties, and adrenergic signaling landscapes, all of which alter drug sensitivity and therapeutic response in ways that standard preclinical models cannot replicate.^11^ The above examples collectively illustrate a systematic pattern in which preclinical in vitro and animal efficacy does not predict clinical outcomes, leading to enormous expenditure of resources on trials that ultimately fail, and in some cases, exposing patients to direct harm. This recognition has intensified the search for disease models that more faithfully reflect human cardiac biology at the cellular, tissue, and physiological levels.

### iPSC-derived in vitro models as transformative platforms

The development of induced pluripotent stem cell (iPSC) technology has fundamentally transformed biomedical research and regenerative medicine.^17^ Unlike embryonic stem cells, iPSCs can be generated from adult somatic cells of individual patients through reprogramming with pluripotency factors, enabling the creation of patient-specific cellular and 3D models without ethical limitations. This capacity for derivation from patients with specific disease mutations, combined with the ability to differentiate iPSCs into nearly any cell type including cardiomyocytes, provides an unprecedented opportunity to create personalized disease models that faithfully recapitulate patient-specific pathophysiology.^18^

## Development and characterization of iPSC-derived cardiac 3D models

### Evolution from 2D to 3D cardiac models

The journey from conventional 2D cardiomyocyte cultures to complex 3D systems represents a fundamental advancement in cardiac disease modeling.^19^ Before describing these systems, it is important to establish a few clear terminological distinctions. A cardiac spheroid refers to a self-assembled, 3D cellular aggregate formed without an exogenous scaffold; while spheroids improve cell-cell contact and paracrine signaling relative to monolayer cultures, they lack defined spatial organization or organ-like architectural patterning.^19^ An engineered cardiac tissue or engineered heart tissue (EHT), by contrast, is a scaffold-based construct in which cells, typically iPSC-derived cardiomyocytes combined with supporting cell types are seeded into a biomaterial matrix or between attachment posts to produce tissues with defined geometry, alignment, and measurable contractile force. These constructs excel for biomechanical characterization and drug testing but require specialized fabrication.^20^ A cardiac organoid occupies a conceptually distinct category: it is a self-organizing, multicellular 3D structure that arises through directed differentiation protocols designed to recapitulate aspects of organ-level architecture and function, including chamber-like morphology, the presence of multiple cardiac lineages (cardiomyocytes, fibroblasts, endothelial cells, smooth muscle cells), and emergent electromechanical behavior that no single cell type produces in isolation.^21^  Table 1 summarizes the key comparative features of these systems. This review provides a synthesis of 3D cardiac systems as disease models for cardiomyopathies, with particular emphasis on cardiac organoids as the primary focus of discussion.

**Table 1:** Context-specific comparison of hiPSC-derived cardiomyocyte culture platforms for cardiomyopathy disease modeling.

Parameter	2D monolayer	3D spheroids	Engineered tissues	Cardiac organoids	Refs.
Cellular complexity	Primarily cardiomyocytes (optional co-culture)	Cardiomyocytes plus stromal cells; limited lineage diversity	Defined multicellular composition	Multilineage and self-organized	^19^ ^,^ ^22–26^
Cell-cell interactions	Limited-moderate	Moderate-high	High	High	^19^ ^,^ ^22–24^ ^,^ ^26^
Tissue-level organization	Low/planar	Intermediate, aggregate-based	High, geometry-controlled	High but variable/self-organized	^23–26^
Oxygen/nutrient diffusion	Uniform access	Radial gradients common	Gradients tunable; perfusion optional	Variable; diffusion limits common	^19^ ^,^ ^23^ ^,^ ^24^ ^,^ ^26^
Electromechanical coupling	Moderate	Moderate	Strong	Moderate-high	^22–24^ ^,^ ^26^ ^,^ ^27^
Arrhythmia modeling	Good for cellular electrophysiology	Moderate-good	Good-excellent	Moderate (improving)	^22^ ^,^ ^24^ ^,^ ^27^
Cost per unit	Low	Low-moderate	High	Moderate-high	^19^ ^,^ ^22–24^ ^,^ ^26^
Throughput	Very high	High	Low-moderate	Low-moderate	^19^ ^,^ ^22^ ^,^ ^24^ ^,^ ^26^ ^,^ ^27^
Maturation status	Low-moderate	Moderate	Moderate-high	Moderate	^22–24^ ^,^ ^27^
Drug screening	Excellent for large-scale screens	Good	Good for mechanistic/functional assays	Moderate	^19^ ^,^ ^22^ ^,^ ^24^ ^,^ ^26^ ^,^ ^27^
Disease recapitulation	Good for cell-autonomous phenotypes	Moderate-good	Good-excellent	High for multicellular/developmental features	^19^ ^,^ ^22–27^
Vascularization	Absent	Absent unless engineered	Limited unless engineered or perfused	Primitive/variable vascular-like networks	^19^ ^,^ ^23–26^

Descriptors are qualitative and protocol dependent. Comparisons are relative and reflect typical implementations reported in peer-reviewed literature rather than absolute performance of any single protocol.

Abbreviations: EHT, engineered heart tissue; hiPSC, human induced pluripotent stem cell.

**Figure 1.**
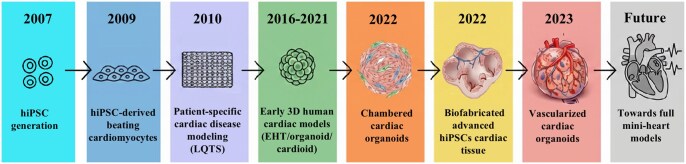
Evolution of iPSC technology derived three-dimensional (3D) cardiac systems for cardiac disease modeling. Historical timeline showing major milestones in human iPSC technology development and cardiac disease modeling using 3D models. Key developments include: (2007) human iPSC (HiPSCs) generation^30^^,^^31^; (2009) HiPSC-derived beating cardiomyocytes^32^^,^^33^; (2010) patient-specific HiPSC cardiac disease-modeling for long QT syndrome^34^; (2016-2021) development of early HiPSC-derived 3D cardiac systems (engineered heart tissue (EHT), organoids and cardioids) and maturation strategies^25^^,^^35–41^; (2022) chambered cardiac organoid generation protocol^42^; (2022) advanced 3D biofabrication and scaffold-based cardiac constructs^43^; (2023) primary research papers for vascularized cardiac organoid^44^^,^^45^; (in future) more advanced engineered 3D cardiac tissues with structural and functional similarities to the human heart.

In Table 1, a detailed comparison of diverse iPSC-derived cardiomyocyte culture systems for cardiomyopathy disease modeling have been made. Early iPSC-derived cardiomyocytes (iPSC-CMs) demonstrated functional properties of adult cardiomyocytes but exhibited significant immaturities including fetal-like electrophysiology, aberrant metabolism, and incomplete sarcomeric organization. The progression toward 3D models emerged from recognition that native cardiac tissue is inherently 3D, with critical spatial organization of multiple cell types, extracellular matrix interactions, and mechanical forces that regulate cellular behavior^28^^,^^29^ (Figure 1).

Two-dimensional monolayer cultures, while enabling detailed cellular characterization through high-resolution microscopy and electrophysiology, lack multicellular interactions and tissue-level architecture. Advancement to 3D spheroid structures improved cellular heterogeneity and electromechanical coupling but still lacked sufficient control over spatial organization.^46^^,^^47^ Engineered heart tissues and cardiac organoids represent the current state of the art, incorporating multiple cell types (cardiomyocytes, fibroblasts, endothelial cells, and smooth muscle cells), defined spatial organization, and often biomechanical stimulation to better recapitulate native cardiac tissue properties^28^^,^^48^^,^^49^ (Figure 1). Interestingly, generated heart-forming organoids co-develop cardiac and foregut endoderm tissues, highlighting the importance of developmental context in recapitulating early human cardiogenesis in vitro.^50^ Extending to this complexity further, multichamber cardioids are capable of modeling distinct ventricular and atrial identities within a single organoid system, revealing previously inaccessible aspects of human heart development and enabling the study of chamber-specific cardiac defects.^51^ More recently, Dardano et al. generated blood-generating heart-forming organoids that co-model the embryonic heart alongside the nascent hematopoietic system, recapitulating the intimate developmental relationship between cardiac morphogenesis and early blood cell formation.^52^ A recent study showed that the co-emergence of cardiac and gut tissues within iPSC-derived organoids promotes cardiomyocyte maturation, suggesting that cross-tissue signaling from adjacent endoderm derivatives is a physiologically important driver of cardiac maturation.^53^ These studies illuminate a great deal of progress in the research of cardiac organoids.

### Strategies for improving maturation of cardiac models

A critical challenge in iPSC-derived cardiac models is the fetal-like phenotype of differentiated cardiomyocytes, which limits their physiological relevance for modeling adult-onset diseases. Cardiomyocyte maturation is a complex, multifaceted process involving sarcomere organization, mitochondrial development, metabolic remodeling, and electrophysiological refinement.^54^^,^^55^ Multiple maturation strategies have been developed to enhance iPSC-CM maturation toward an adult phenotype (Table S1).

Metabolic maturation protocols leverage the observation that adult cardiomyocytes predominantly utilize oxidative metabolism of fatty acids, whereas immature iPSC-CMs exhibit predominantly glycolytic metabolism (Table S1). Exposure to fatty acid substrates, hormonal factors such as thyroid hormone and glucocorticoids, and metabolic modulators including mTOR inhibitors have been shown to enhance mitochondrial maturation and metabolic sophistication.^56^ Mechanical stimulation through electrical pacing, mechanical stretching, or 3D tissue engineering approaches promotes sarcomeric organization and functional contractile properties properties.^28^ Co-culture with cardiac fibroblasts and other supporting cells enhances maturation through paracrine signaling and provides critical structural support.^57^ Novel approaches using specific growth factors such as Sfrp2 have been demonstrated to induce robust cardiac differentiation and maturation^58^ (Table S1).

### Multicellular architecture and function

Contemporary cardiac 3D systems incorporate multiple cell types that self-organize into tissue-like structures mimicking native cardiac tissue organization and function. The inclusion of fibroblasts, endothelial cells, and smooth muscle cells alongside cardiomyocytes recapitulates essential cellular interactions and enables investigation of cell-cell communication in disease states.^59^^,^^60^ Multicellular organoids demonstrate improved electromechanical coupling, calcium handling, and contractile properties compared to cardiomyocyte-only systems.^61^

Recent advances in high-throughput organoid generation in stirred suspension bioreactors have enabled scalable production of cardiac organoids with improved batch-to-batch consistency and yield.^62^ These bioreactor-based approaches produce organoids with approximately 94% purity, high postcryopreservation viability exceeding 90%, and predominantly ventricular identity.^62^ The reproducibility and scalability of these systems are critical for translating organoid-based drug screening and disease modeling into widespread clinical and pharmaceutical applications.^62^

## Disease modeling, therapeutic applications, and drug discovery

### Disease modeling for genetic cardiomyopathies

Induced pluripotent stem cell models of inherited cardiomyopathies have been used to reveal mechanisms beyond the reach of animal models or conventional cell lines. Hypertrophic cardiomyopathy (HCM), the most common inherited cardiac disorder affecting approximately 1 in 500 individuals worldwide, is caused predominantly by mutations in genes encoding sarcomeric proteins, particularly MYH7 and MYBPC3.^63^ Induced pluripotent stem cell-derived models of HCM have successfully recapitulated key disease features including cellular hypertrophy, sarcomeric disarray, altered calcium handling, and electrophysiological abnormalities^64^ (Table S2). Organoid models derived from patients with MYH7 mutations demonstrate increased cell size, enhanced contractility, and arrhythmogenic behaviors that mirror clinical observations and have enabled evaluation of myosin inhibitors and gene editing strategies that reduce hypertrophic phenotypes.^65^^,^^66^

Dilated cardiomyopathy, characterized by ventricular enlargement and systolic dysfunction, has a genetic basis in approximately 20%-35% cases,^67^ with mutations in TTN (titin), LMNA (lamin A/C), and RBM20 among the most frequently implicated.^67^ Patient-specific iPSC models have revealed mechanistically distinct pathological pathways: TTN truncation variants impair sarcomere assembly and increase susceptibility to stress-induced damage^68^; LMNA mutations produce nuclear envelope abnormalities, altered gene expression, and both contractile dysfunction and arrhythmias^69^; and RBM20 mutations affect alternative splicing of multiple cardiac genes, leading to aberrant protein isoforms and compromised cardiac function^70^ (Table S2).

Transthyretin amyloid cardiomyopathy (ATTR-CM) is a rare but severe cause of restrictive cardiomyopathy, characterized by myocardial accumulation of transthyretin amyloid fibrils. It occurs in both hereditary forms caused by pathogenic TTR variants and wild-type, age-associated forms, and represents an additional disease setting in which cardiac disease modeling can provide mechanistic insight.^71^ A liver-heart microphysiological organoid model has been used to simulate hepatic production of pathogenic TTR-Val122Ile and downstream cardiac exposure, reproducing features such as oxidative stress, protein aggregation, impaired relaxation, abnormal calcium handling, and conduction defects.^72^^,^^73^ Complementary iPSC-based ATTR-CM models show that wild-type and variant TTR fibrils induce cell-type-specific injury in cardiomyocytes, endothelial cells, and fibroblasts, including sarcomere disruption, altered calcium handling, impaired electromechanical coupling, endothelial dysfunction, and fibroblast-fibril interactions.^74^ These studies support the potential of ATTR-CM cardiac organoids to model amyloid deposition, multicellular myocardial toxicity, and therapeutic rescue.

Across cardiomyopathy subtypes, iPSC-based 3D models combine patient-specific genetics with 3D tissue organization and functional readouts to reveal tissue-level disease mechanisms and test therapies in a human-relevant context. Together, these platforms represent a transformative advance over earlier models and lay the foundation for future therapeutic applications.

### Drug screening and efficacy testing

Human cardiac 3D models, especially cardiac organoids, provide a more physiologically relevant platform for drug screening than conventional 2D systems because they preserve multicellular architecture, spontaneous beating, and coupled electrophysiological and mechanical readouts, while also revealing toxicity phenotypes that are harder to detect in simpler cultures.^59^^,^^75^ A recent work using an advanced cardiac organoid model showed that doxorubicin exposure reduced organoid size and increased collagen deposition, consistent with a robust cardiotoxicity phenotype.^76^ Comparable findings were reported for maprotiline. In hiPSC-derived, self-assembled cardiac organoids, the drug induced developmental cardiotoxicity, and the authors directly contrasted these effects with mature cardiac organoids to highlight stage specific vulnerability.^77^ Sertraline produced a similar developmental toxicity signal in neuro cardiac assembloids, where it increased beating rate and resulted in less developed neurites. Together, these studies underscore how organoid and assembloid platforms can resolve both mature and developmental cardiotoxicity mechanisms.^78^

Several disease-specific cardiomyopathies organoid models are being employed to evaluate various potential treatment strategies. For example, HCM organoids have been used to evaluate myosin inhibitors, demonstrating dose-dependent reduction in cellular hypertrophy and normalization of contractile function.^75^^,^^79^ Dilated cardiomyopathy organoids have facilitated testing of gene therapy approaches, including delivery of functional gene copies or exon-skipping strategies for truncation mutations.^80^ The ability to derive organoids from individual patients theoretically enables personalized drug screening, although practical implementation requires further optimization of throughput and cost-effectiveness.^59^

### Gene editing and correction strategies

The combination of iPSC technology with CRISPR-Cas9 gene editing offers transformative possibilities to produce 3D cardiac models for both disease modeling and potential therapeutic intervention. Three-dimensional tissues derived from isogenic pairs of iPSC lines enable rigorous mechanistic studies by eliminating confounding genetic background effects. Furthermore, gene correction in patient-derived iPSCs can restore normal cellular phenotypes, validating causative mutations and demonstrating proof-of-principle for genetic therapies.^81^^,^^82^ Several studies have successfully corrected disease-causing mutations in cardiomyopathy iPSC lines, resulting in normalization of cellular phenotypes in subsequently derived organoids. For example, correction of MYH7 mutations in HCM iPSCs eliminates cellular hypertrophy and contractile abnormalities.^83^ Similarly, correction of LMNA mutations in DCM models restores nuclear envelope structure and improves contractile function.^84^ While technical challenges remain for in vivo gene editing, including efficient delivery of editing machinery to the heart, off-target editing events, incomplete editing efficiency across cardiac cell populations, immune responses to viral vectors, and the difficulty of editing post-mitotic cardiomyocytes in situ, these studies establish the potential for curative genetic therapies and provide valuable tools for therapeutic development.

## Technical challenges and future directions

### Maturation and physiological fidelity

Despite significant progress in maturation strategies, iPSC-derived cardiomyocytes typically retain a fetal-like phenotype that limits their utility for modeling adult-onset diseases. Key maturation markers including sarcomere structure, metabolic profile, electrophysiological properties, and force generation remain intermediate between fetal and adult cardiomyocytes.^29^^,^^85^ This developmental limitation affects both disease phenotype manifestation and drug response prediction. Future advances may address this challenge through combination approaches integrating metabolic, mechanical, and electrical stimulation with defined culture conditions and prolonged maturation times. Novel strategies including biomaterial engineering to mimic native extracellular matrix, advanced bioreactor systems providing appropriate mechanical and electrical cues, and co-culture with supporting cell types show promise for enhancing maturation.^29^^,^^86^ Additionally, direct reprogramming approaches that bypass the pluripotent state might generate more mature cardiomyocytes, although this remains an active area of investigation.

### Vascularization, tissue engineering, and chamber architecture

A critical limitation of current cardiac organoids is the lack of functional vasculature. Native cardiac tissue is highly vascularized, with capillaries adjacent to every cardiomyocyte to support the intense metabolic demands of contractile function (Table 1). In the absence of perfusion, organoids rely on diffusion for nutrient and oxygen supply, limiting achievable tissue thickness to approximately 100-200 µm and creating hypoxic cores in larger constructs.^87^^,^^88^ Multiple strategies are being developed to address this limitation. Co-culture with endothelial cells promotes formation of primitive vascular networks within organoids, although these structures typically lack perfusion and hierarchical organization.^88^^,^^89^ Bioprinting approaches enable precise spatial patterning of different cell types to create vascular channels, and some groups have demonstrated perfusion of engineered cardiac tissues in bioreactor systems.^88^^,^^89^ Integration with host vasculature following implantation remains a challenge, with research focused on promoting angiogenesis and anastomosis with recipient vessels. Organ-on-a-chip platforms with integrated microfluidic perfusion provide an alternative approach, enabling nutrient delivery and waste removal while maintaining 3D tissue architecture.^90^ Cruz-Moreira et al. developed a heart-on-chip system with an integrated peristaltic pump enabling precise, automated flow control around 3D cardiac tissues, allowing assessment of how different perfusion conditions affect maturation and mechanotransduction.^91^ Beyond standard channel perfusion, another study demonstrated a beating, microvascularized heart-on-a-chip by combining iPSC-derived cardiomyocyte spheroids with endothelial and fibroblast cells within a vascularized organ-on-chip, enabling evaluation of drug safety under physiologically relevant intravascular delivery conditions.^92^

An important limitation of current cardiac 3D tissue systems is that they do not fully recapitulate the native 4-chambered architecture and valve-enforced hemodynamic environment of the heart, and thus only partially capture spatially defined heterogeneity.^93^ Another limitation to these 3D models is that the extracellular matrix within engineered constructs remains developmentally immature, with simplified composition and altered stiffness compared to adult myocardium, which in turn constrains full structural and functional maturation of cardiomyocytes.^49^^,^^94^

### Standardization and reproducibility

For cardiac organoid technology to achieve its translational potential, improved standardization and reproducibility across laboratories are essential. Current organoid generation protocols vary significantly in differentiation methods, cell line characteristics, culture conditions, and analysis approaches, leading to substantial variability in results.^95^^,^^96^ This variability is compounded by inconsistent use of terminology: what one group calls an “organoid” may be classified as a “cardiac microtissue” or “cardiac spheroid” by another, making cross-study comparisons particularly challenging, thereby hindering regulatory approval for clinical applications.

Efforts to address this challenge include development of quality control metrics, standardized protocols, and reference materials for iPSC-derived cardiac cells. Consortia such as the Human Cell Atlas and International Stem Cell Banking Initiative are working to establish standards for cell characterization, culture methods, and data reporting.^97^^,^^98^ Advanced analytical approaches including machine learning for automated phenotyping and quality assessment may improve consistency. Furthermore, commercial availability of validated iPSC lines and differentiation reagents is increasing accessibility and reducing variability.^99–101^

## Translational perspective and clinical impact

### From bench to bedside: current status and future vision

The translational path from iPSC-derived cardiac organoids to clinical application encompasses multiple stages, each with distinct challenges and opportunities. In the near term, organoid models are primarily impacting drug discovery and development pipelines, providing more predictive preclinical tools that may reduce costly clinical trial failures. Several pharmaceutical companies have integrated iPSC-derived cardiac models into their screening platforms for both efficacy and cardiotoxicity assessment.^102^^,^^103^

Medium-term applications include precision medicine approaches where patient-derived organoids inform treatment selection. For patients with rare genetic cardiomyopathies or unclear diagnoses, functional testing in personalized organoid models could guide therapeutic decisions. This vision requires further advances in organoid generation efficiency, functional analysis automation, and validation of predictive accuracy. Successful implementation would represent a paradigm shift toward individualized cardiovascular care.^79^^,^^104^

Long-term possibilities include curative genetic therapies and regenerative cell therapies. As gene editing technologies mature and safety profiles are established, correction of disease-causing mutations in patient cells followed by reimplantation could potentially cure genetic cardiomyopathies. Similarly, cell-based therapies using iPSC-derived cardiomyocytes or engineered tissues might restore function in damaged or failing hearts. While significant technical and regulatory hurdles remain, early phase clinical trials are beginning to explore these possibilities.^82^^,^^105–107^

### Regulatory and ethical considerations

As iPSC-derived cardiac models and therapies advance toward clinical application, appropriate regulatory frameworks must be established. Regulatory agencies including the U.S. Food and Drug Administration (FDA) and European Medicines Agency (EMA) are developing guidelines for stem cell-derived products, addressing issues including cell line characterization, manufacturing standards, quality control, and safety assessment.^108–110^ The complexity and novelty of these products require careful consideration of existing regulatory paradigms and adaptation where necessary.

Ethical considerations include informed consent processes for cell derivation, appropriate use of genetic information, equity of access to personalized medicine approaches, and responsible conduct of early phase clinical trials. As these technologies become more accessible, international consensus on ethical guidelines will be essential. Patient advocacy groups, bioethicists, regulators, and researchers must collaborate to ensure responsible development and deployment of these transformative technologies.^111–113^

## Summary and outlook

Induced pluripotent stem cell-derived cardiac organoids represent a transformative platform for cardiovascular research, bridging the critical gap between preclinical models and human disease. These sophisticated 3D tissue models provide unprecedented opportunities to investigate cardiomyopathy mechanisms, screen therapeutic candidates, and advance toward personalized medicine. While significant technical challenges remain, particularly regarding cellular maturation, vascularization, and standardization, rapid progress is being made through interdisciplinary collaboration integrating stem cell biology, bioengineering, and computational approaches. The integration of organoid technology with gene editing, advanced imaging, and computational modeling promises to accelerate understanding of cardiovascular disease and therapeutic development. As these technologies mature and become more accessible, their impact will extend beyond research laboratories to clinical practice, potentially revolutionizing how we diagnose, understand, and treat cardiac diseases. The ultimate vision of true precision medicine, where patient-specific organoids inform individualized therapeutic strategies is becoming increasingly achievable, heralding a new era in cardiovascular medicine.

## Supplementary Material

sxag039_Supplementary_Data

## Data Availability

This article is a review of previously published studies. No new datasets were generated or analyzed during the current study.
