# Supplementary material for: Human iPSC-derived 3D cardiac models for cardiomyopathies: organoids, spheroids, and engineered heart tissues as translational platforms
Source: Stem Cells. 2026 Jul 8;44(8):sxag039. doi: 10.1093/stmcls/sxag039 (PMC13452171; doi:10.1093/stmcls/sxag039)
Supplement: Supplementary file 1 [file sxag039_supplementary_data.docx]

**Supplementary Information**

**Human iPSC-Derived 3D Cardiac Models for Cardiomyopathies: Organoids, Spheroids, and Engineered Heart Tissues as Translational Platforms**

Dr. Prabin Upadhyaya, M.Sc., Ph.D.^1,2^

^1^ Division of Cardiology, Department of Medicine and Surgery, Università degli studi di Milano-Bicocca, Milan, Italy

^2^ Experimental Laboratory for Genetic-Based Cardiac Arrhythmia Research, IRCCS Istituto Auxologico Italiano, Cusano Milanino (MI), Italy.

**Corresponding Author**

Dr. Prabin Upadhyaya, Ph.D.

Division of Cardiology, Department of Medicine and Surgery

Università degli studi di Milano-Bicocca

Piazza dell'Ateneo Nuovo, 1, 20126, Milan, Italy

Experimental Laboratory for Genetic-Based Cardiac Arrhythmia Research

IRCCS Istituto Auxologico Italiano,

Via Zucchi 18, 20095 Cusano Milanino, Milan, Italy.

Tel: +39 351 532 8661

Email: [prabin.upadhyaya@unimib.it](mailto:prabin.upadhyaya@unimib.it)

| **Maturation Strategy** | **Model Type** | **Mechanism** | **Functional Outcome** | **Timeframe** | **Ref.** |
| --- | --- | --- | --- | --- | --- |
| **Metabolic Maturation** | Cardiomyocytes | Fatty acid substrates + thyroid hormone | ↑ Oxidative metabolism,  ↑ mitochondrial function | 20-30 days | [1,2] |
| **Mechanical Stimulation** | EHT | Electrical pacing + cyclic stretch | ↑ Sarcomere organization,  ↑ contractile force | 14-28 days | [3,4] |
| **Co-culture** | Spheroids, Organoids | Cardiac fibroblasts + endothelial cells | ↑ Maturation markers,  ↑ cell-cell coupling | 14-21 days | [5,6] |
| **3D Architecture** | EHT, Organoids | Engineered tissues + ECM | ↑ Tissue organization,  ↑ electromechanical coupling | 21-35 days | [7,8] |
| **Long-Term Culture** | Cardiomyocytes | Extended culture >60 days+ T3/T4 hormones+ glucose-to-fatty acid media switch | ↑ Sarcomere length,  ↑ t-tubule formation,  ↑ MYH7/MYH6 ratio,  ↑ Adult-like Ca2+ handling | 60-120 days | [9,10] |
| **Substrate Stiffness Modulation** | Cardiomyocytes, EHT | Tunable hydrogels (polyacrylamide, PDMS, GelMA) mimicking cardiac ECM (~10 kPa) | ↑ Sarcomere organization,  ↑ Contractile force,  ↑ YAP/TAZ mechanosignaling | 14-30 days | [11–15] |
| **Microfluidic Perfusion Systems** | Organoids, EHT | Continuous nutrient/O2 delivery+ shear stress+ organ-on-chip platforms | ↑ Cell viability,  ↑ Metabolic activity,  ↑ Electromechanical maturation,  ↑ Drug response fidelity | 21-60 days | [16–19] |

**Table S1. Strategies for functional maturation of iPSC-derived cardiomyocytes, spheroids, EHTs and cardiac organoids.** This table summarizes key experimental approaches used to enhance maturation of iPSC-derived cardiac models, including metabolic modulation, mechanical stimulation, co-culture systems, three-dimensional architectural design, long-term culture paradigms, substrate stiffness tuning, and microfluidic perfusion platforms. For each strategy, representative studies are synthesized with respect to model type, underlying mechanistic basis, and resulting functional outcomes such as electrophysiological properties, calcium handling, contractility, and structural organization. Time-dependent effects on maturation are also highlighted to provide a comparative framework for evaluating protocol efficiency and translational relevance.

| **Disease** | **Primary Gene(s)** | **Mutation Type** | **Key Phenotypes** | **Prevalence (% of cases)** | **Organoid Findings** | **Ref.** |
| --- | --- | --- | --- | --- | --- | --- |
| **HCM** | MYH7 | Missense | Hypertrophy, Disarray, Arrhythmia | 40%–50% of genotype-positive HCM cases | ↑APD90, ↑MYH7 | [20–22] |
| **HCM** | MYBPC3 | Frameshift/ Missense | Hypertrophy, Mitochondrial dysfunction | 35%–40% of genotype-positive HCM cases | ↑NPPA, ↓contractility | [22,23] |
| **DCM** | TTN | Truncating | Reduced contractility, Disarray | TTNtv is identified in ~11-15% of adult cases of sporadic DCM; ~23-27% cases of familial DCM. | ↓contractile force, ↑arrhythmias | [24,25] |
| **DCM** | LMNA | Frameshift/ Missense | Nuclear abnormalities, Arrhythmias | ~6–8% of familial DCM cases, ~5–13% cases of DCM of unknown cause/ idiopathic DCM | ↓APD90, ↑contractility | [26–28] |
| **DCM** | RBM20 | Missense | Splicing defects, Contractile dysfunction | ~3% in DCM cohorts, ~2-6% of familial DCM cases | Abnormal splicing, ↓contractility | [29–32] |
| **Amyloid CM** | TTR | Missense | Proteotoxicity, Sarcomeric disarray | 100% of hereditary ATTR-CM (ATTRv-CM) cases are caused by pathogenic TTR variants; ATTR-CM diagnosed patients possess a pathogenic TTR mutation in 43.2% of females and 17.3% of males. | ↑Oxidative stress, ↑Apoptosis | [33–36] |

**Table S2. Key gene mutations and disease modelling in iPSC-derived cardiac organoids.** The table provides an overview of major cardiomyopathies, the principal disease-associated genes and mutation types, their prevalence among affected individuals, and the resulting cellular and functional phenotypes. It further highlights studies employing iPSC-derived cardiac organoid models and summarizes the key mechanistic insights and disease-relevant findings generated from these systems.

**References:**

[1]	Guo Y, Pu WT. Cardiomyocyte Maturation: New Phase in Development. Circ Res 2020;126:1086. https://doi.org/10.1161/CIRCRESAHA.119.315862.

[2]	Correia C, Christoffersson J, Tejedor S, El-Haou S, Matadamas-Guzman M, Nair S, et al. Enhancing Maturation and Translatability of Human Pluripotent Stem Cell-Derived Cardiomyocytes through a Novel Medium Containing Acetyl-CoA Carboxylase 2 Inhibitor. Cells 2024;13:1339. https://doi.org/10.3390/cells13161339.

[3]	Ruan JL, Tulloch NL, Razumova M V., Saiget M, Muskheli V, Pabon L, et al. Mechanical Stress Conditioning and Electrical Stimulation Promote Contractility and Force Maturation of Induced Pluripotent Stem Cell-Derived Human Cardiac Tissue. Circulation 2016;134:1557–67. https://doi.org/10.1161/CIRCULATIONAHA.114.014998.

[4]	Li J, Zhang L, Yu L, Minami I, Miyagawa S, Hörning M, et al. Circulating re-entrant waves promote maturation of hiPSC-derived cardiomyocytes in self-organized tissue ring. Communications Biology 2020 3:1 2020;3:122-. https://doi.org/10.1038/s42003-020-0853-0.

[5]	Giacomelli E, Meraviglia V, Campostrini G, Cochrane A, Cao X, van Helden RWJ, et al. Human-iPSC-Derived Cardiac Stromal Cells Enhance Maturation in 3D Cardiac Microtissues and Reveal Non-cardiomyocyte Contributions to Heart Disease. Cell Stem Cell 2020;26:862-879.e11. https://doi.org/10.1016/j.stem.2020.05.004.

[6]	Beauchamp P, Jackson CB, Ozhathil LC, Agarkova I, Galindo CL, Sawyer DB, et al. 3D Co-culture of hiPSC-Derived Cardiomyocytes With Cardiac Fibroblasts Improves Tissue-Like Features of Cardiac Spheroids. Front Mol Biosci 2020;7:505658. https://doi.org/10.3389/fmolb.2020.00014.

[7]	Fullenkamp DE, Maeng WY, Oh S, Luan H, Kim KS, Chychula IA, et al. Simultaneous electromechanical monitoring in engineered heart tissues using a mesoscale framework. Sci Adv 2024;10:7089. https://doi.org/10.1126/sciadv.ado7089.

[8]	Tzatzalos E, Abilez OJ, Shukla P, Wu JC. Engineered heart tissues and induced pluripotent stem cells: macro- and microstructures for disease modeling, drug screening, and translational studies. Adv Drug Deliv Rev 2015;96:234. https://doi.org/10.1016/j.addr.2015.09.010.

[9]	Dai DF, Danoviz ME, Wiczer B, Laflamme MA, Tian R. Mitochondrial Maturation in Human Pluripotent Stem Cell Derived Cardiomyocytes. Stem Cells Int 2017;2017:5153625. https://doi.org/10.1155/2017/5153625.

[10]	Karbassi E, Fenix A, Marchiano S, Muraoka N, Nakamura K, Yang X, et al. Cardiomyocyte maturation: advances in knowledge and implications for regenerative medicine. Nat Rev Cardiol 2020;17:341. https://doi.org/10.1038/S41569-019-0331-X.

[11]	Shahannaz DC, Sugiura T. Engineering Smart Biomaterial Interfaces for iPSC-CM Maturation: A Biophysical and Metabolic Reprogramming Approach to Regenerative Cardiac Medicine. International Journal of Molecular Sciences 2026, Vol 27, 2026;27. https://doi.org/10.3390/IJMS27062637.

[12]	Pardon G, Vander Roest AS, Chirikian O, Birnbaum F, Lewis H, Castillo EA, et al. Tracking single hiPSC-derived cardiomyocyte contractile function using CONTRAX an efficient pipeline for traction force measurement. Nature Communications 2024 15:1 2024;15:5427-. https://doi.org/10.1038/s41467-024-49755-3.

[13]	Vinarsky V, Pagliari S, Aldabash B, Martino F, Mazzotti C, Jirakova K, et al. YAP1 reactivation in cardiomyocytes following ECM remodelling contributes to the development of contractile force and sarcomere maturation. Cell Death Discovery 2025 11:1 2025;11:518-. https://doi.org/10.1038/s41420-025-02793-2.

[14]	Bhana B, Iyer RK, Chen WLK, Zhao R, Sider KL, Likhitpanichkul M, et al. Influence of substrate stiffness on the phenotype of heart cells. Biotechnol Bioeng 2010;105:1148–60. https://doi.org/10.1002/BIT.22647.

[15]	Herron TJ, Da Rocha AM, Campbell KF, Ponce-Balbuena D, Willis BC, Guerrero-Serna G, et al. Extracellular matrix-mediated maturation of human pluripotent stem cell-derived cardiac monolayer structure and electrophysiological function. Circ Arrhythm Electrophysiol 2016;9. https://doi.org/10.1161/CIRCEP.113.003638;WEBSITE:WEBSITE:AHA-SITE;REQUESTEDJOURNAL:JOURNAL:CIRCEP;JOURNAL:JOURNAL:CIRCEP;WGROUP:STRING:PUBLICATION.

[16]	Marsano A, Conficconi C, Lemme M, Occhetta P, Gaudiello E, Votta E, et al. Beating heart on a chip: a novel microfluidic platform to generate functional 3D cardiac microtissues. Lab Chip 2016;16:599–610. https://doi.org/10.1039/C5LC01356A.

[17]	Khosravi R, Radisic M. Heart-on-a-chip and vasculature-on-a-chip platforms as models of cardiovascular disease. Nature Reviews Cardiology 2026 2026:1–18. https://doi.org/10.1038/s41569-026-01255-1.

[18]	Kim YH, Son YH, Choi Y, Kim MS, Park SJ, Lee KY. Advanced in vitro cardiac models for drug evaluation: integration of organoids, engineered tissues, and microphysiological systems. Microsystems & Nanoengineering 2026 12:1 2026;12:162-. https://doi.org/10.1038/s41378-026-01249-6.

[19]	Menzani B, De Gea P, Gidrol X, Tubbs E. Lab on a Chip CRITICAL REVIEW Vascularizing organoids-on-chip for perfused and personalized models 2026. https://doi.org/10.1039/d5lc00890e.

[20]	Han L, Li Y, Tchao J, Kaplan AD, Lin B, Li Y, et al. Study familial hypertrophic cardiomyopathy using patient-specific induced pluripotent stem cells. Cardiovasc Res 2014;104:258–69. https://doi.org/10.1093/cvr/cvu205.

[21]	Wang K, Schriver BJ, Aschar-Sobbi R, Yi AY, Feric NT, Graziano MP. Human engineered cardiac tissue model of hypertrophic cardiomyopathy recapitulates key hallmarks of the disease and the effect of chronic mavacamten treatment. Front Bioeng Biotechnol 2023;11. https://doi.org/10.3389/fbioe.2023.1227184.

[22]	Lopes LR, Ho CY, Elliott PM. Genetics of hypertrophic cardiomyopathy: established and emerging implications for clinical practice. Eur Heart J 2024;45:2727–34. https://doi.org/10.1093/EURHEARTJ/EHAE421.

[23]	Desai D, Song T, Singh RR, Baby A, McNamara J, Green LC, et al. MYBPC3 D389V Variant Induces Hypercontractility in Cardiac Organoids. Cells 2024;13:1913. https://doi.org/10.3390/cells13221913.

[24]	Huang G, Bisaria A, Wakefield DL, Yamawaki TM, Luo X, Zhang JA, et al. Titin-truncating variants in hiPSC cardiomyocytes induce pathogenic proteinopathy and sarcomere defects with preserved core contractile machinery. Stem Cell Reports 2022;18:220. https://doi.org/10.1016/j.stemcr.2022.11.008.

[25]	Newman NA, Burke MA. Dilated Cardiomyopathy: A Genetic Journey from Past to Future. International Journal of Molecular Sciences 2024, Vol 25, 2024;25. https://doi.org/10.3390/IJMS252111460.

[26]	Wallace M, Zahr H, Perati S, Morsink CD, Johnson LE, Gacita AM, et al. Nuclear damage in LMNA mutant iPSC-derived cardiomyocytes is associated with impaired lamin localization to the nuclear envelope. Mol Biol Cell 2023;34:ar113. https://doi.org/10.1091/mbc.E21-10-0527.

[27]	Vaikhanskaya T, Sivitskaya L, Danilenko N, Davydenko O, Kurushka T, Sidorenko I. LMNA-Related Dilated Cardiomyopathy. Oxf Med Case Reports 2022;2014:102–4. https://doi.org/10.1093/omcr/omu040.

[28]	Crasto S, My I, Di Pasquale E. The Broad Spectrum of LMNA Cardiac Diseases: From Molecular Mechanisms to Clinical Phenotype. Front Physiol 2020;11:538082. https://doi.org/10.3389/FPHYS.2020.00761/TEXT.

[29]	Briganti F, Sun H, Wei W, Wu J, Zhu C, Liss M, et al. iPSC Modeling of RBM20-Deficient DCM Identifies Upregulation of RBM20 as a Therapeutic Strategy. Cell Rep 2020;32:108117. https://doi.org/10.1016/j.celrep.2020.108117.

[30]	Fenix AM, Miyaoka Y, Bertero A, Blue SM, Spindler MJ, Tan KKB, et al. Gain-of-function cardiomyopathic mutations in RBM20 rewire splicing regulation and re-distribute ribonucleoprotein granules within processing bodies. Nature Communications 2021 12:1 2021;12:6324-. https://doi.org/10.1038/s41467-021-26623-y.

[31]	Carigi S, Olivucci G, Cristalli CP, Marzo F, Isidori F, Palmieri S, et al. A single RBM20 missense variant is a potential contributor to dilated cardiomyopathy and/or isolated left ventricular dilatation in the Emilia Romagna region of Italy. Int J Cardiol 2025;423:132999. https://doi.org/10.1016/J.IJCARD.2025.132999.

[32]	Martini M, Bueno Marinas M, Rigato I, Pilichou K, Bauce B. Clinical Insights in RNA-Binding Protein Motif 20 Cardiomyopathy: A Systematic Review. Biomolecules 2024, Vol 14, 2024;14. https://doi.org/10.3390/BIOM14060702.

[33]	Liu Y, Riaz M. Human-Induced Pluripotent Stem Cell Models for Amyloid Cardiomyopathy: From Mechanistic Insights to Therapeutic Discovery. Journal of Cardiovascular Development and Disease 2025, Vol 12, Page 434 2025;12:434. https://doi.org/10.3390/jcdd12110434.

[34]	Qin J, Qiu Z, Fan Y, Xiong Q, Lei Z, Wei J, et al. In vitro and in vivo disease models of cardiac amyloidosis: progress, pitfalls, and potential. Cardiovasc Res 2025;121:1997–2013. https://doi.org/10.1093/cvr/cvaf152.

[35]	Porcari A, Razvi Y, Masi A, Patel R, Ioannou A, Rauf MU, et al. Prevalence, Characteristics and Outcomes of Older Patients with Hereditary Versus Wild-Type Transthyretin Amyloid Cardiomyopathy. Eur J Heart Fail 2023;25:515–24. https://doi.org/10.1002/EJHF.2776.

[36]	Ruberg FL, Grogan M, Hanna M, Kelly JW, Maurer MS. Transthyretin Amyloid Cardiomyopathy: JACC State-of-the-Art Review. J Am Coll Cardiol 2019;73:2872–91. https://doi.org/10.1016/J.JACC.2019.04.003.
